# Open Access & ecancermedicalscience

**DOI:** 10.3332/ecancer.2007.ed1

**Published:** 2007-09-18

**Authors:** Umberto Veronesi

**Affiliations:** Founding Editor of ecancermedicalscience, European Institute of Oncology, Milan, Italy

There is a general feeling of dissatisfaction about the way we can inform the scientific community on our discoveries and the results of our studies. The first problem is the long time required before a paper is published. After we spend a relatively short time preparing a paper, the time for acceptance or refusal is often several months, after which if accepted and duly corrected, many more months or even an entire year is needed to see the paper published. All this is certainly not in favour of a rapid dissemination of information and may slow down scientific progress.

The second issue refers to the availability of scientific information to all continents and to remote corners of the world. This is the Galleon concept of Universalism of science, which is not presently fulfilled. The main obstacles are the high cost of journal subscriptions and the problems of distributing paper journals to small cities in developing countries.

The third drawback of the present system lies in the fact that any comment, criticism or objection to any paper requires weeks or months to be published and the same for the author’s reply.

Personally I believe that the best method, which may guarantee a systematic diffusion worldwide, is electronic distribution. The number of computers worldwide, is increasingly exponentially every year. There is a project, by Negroponte known as ‘one laptop per child’ that in the future every citizen of the world will have access to a computer. This great project is to produce an enormous number of personal computers at a very low cost ($100) for children in the developing world. In a world which is progressing electronically the distribution of scientific information via a paper journal appears old fashioned and the change to an electronic network offers great advantages.

We at The European Institute of Oncology have developed a project for the online dissemination of new oncological scientific knowledge. We are launching a novel online journal ecancermedicalscience, where e stands for electronic but also for European. The journal aims to reduce the main obstacles which I mentioned at the beginning ecancermedicalscience will provide immediate workup of received papers with acceptance or refusal in one week. If the paper is accepted it will be published online within one month. Comments, criticisms and objections may go on line immediately after the appearance of the paper. This will open up a system of extensive discussion among the members of the scientific community, which at present is not always possible.

Publication will be free of charge. The consultation of the papers will be free of charge. The participation to the discussion will be free of charge. In other words the journal will be free for the authors and free for the readers.

I am also very excited by the prospect of ecancertv. Here you will all be able to submit, watch and discuss innovative new procedures. Presently you can view an operation on ovarian cancer performed at the European Institute of Oncology where robotic surgery techniques are displayed for all to see. Eventually I hope this feature will enable all those involved in treating cancer patients the chance to view the latest techniques and benefit from the expertise of others. Ultimately we all want to share knowledge so that patients can receive the latest, optimum treatment.

I am very pleased that ecancermedicalscience is the scientific and multimedia partner of The European CanCer Organisation (ECCO). I was associated with ECCO (formally FECS) from the very beginning and their support on this project has been invaluable.

Finally ecancermedicalscience is not for profit, ensuring an unbiased approach to publishing and editing, free from commercial constraints. All the charitable partners involved in the organisation benefit from the success of ecancermedicalscience.

